# Association between Salivary Leptin Levels and Taste Perception in Children

**DOI:** 10.1155/2017/7260169

**Published:** 2017-07-24

**Authors:** Lénia Rodrigues, Rosa Espanca, Ana Rodrigues Costa, Célia Miguel Antunes, Clarinda Pomar, Fernando Capela-Silva, Cristina Conceição Pinheiro, Francisco Amado, Elsa Lamy

**Affiliations:** ^1^Institute of Mediterranean Agricultural and Environmental Sciences (ICAAM), IIFA, University of Évora, Évora, Portugal; ^2^Grouping of Health Centers of Alentejo (ACES-Central), Évora, Portugal; ^3^Institute of Earth Sciences (ICT), Institute of Mediterranean Agricultural and Environmental Sciences (ICAAM) and Department of Chemistry, University of Évora, Évora, Portugal; ^4^Centre of Research in Education and Psychology (CIEP) and Department of Pedagogy, University of Évora, Évora, Portugal; ^5^Institute of Mediterranean Agricultural and Environmental Sciences (ICAAM) and Department of Biology, University of Évora, Évora, Portugal; ^6^Institute of Mediterranean Agricultural and Environmental Sciences (ICAAM) and Department of Zootechnics, University of Évora, Évora, Portugal; ^7^Organic Chemistry, Natural and Agrofood Products (QOPNA) and Department of Chemistry, University of Aveiro, Aveiro, Portugal; ^8^Institute of Mediterranean Agricultural and Environmental Sciences (ICAAM), Núcleo da Mitra, Universidade de Évora, 7006-554 Évora, Portugal

## Abstract

The satiety inducing hormone leptin acts not only at central nervous system but also at peripheral level. Leptin receptors are found in several sense related organs, including the mouth. A role of leptin in sweet taste response has been suggested but, until now, studies have been based on in vitro experiments, or in assessing the levels of the hormone in circulation. The present study investigated whether the levels of leptin in saliva are related to taste perception in children and whether Body Mass Index (BMI) affects such relationship. Sweet and bitter taste sensitivity was assessed for 121 children aged 9-10 years and unstimulated whole saliva was collected for leptin quantification, using ELISA technique. Children females with lower sweet taste sensitivity presented higher salivary leptin levels, but this is only in the normal weight ones. For bitter taste, association between salivary leptin and caffeine threshold detection was observed only in preobese boys, with higher levels of salivary hormone in low sensitive individuals. This study is the first presenting evidences of a relationship between salivary leptin levels and taste perception, which is sex and BMI dependent. The mode of action of salivary leptin at taste receptor level should be elucidated in future studies.

## 1. Introduction

Leptin is a hormone primarily produced by white adipose tissue that regulates energy homeostasis by inducing satiety and energy expenditure [1]. This 16 kDa protein is encoded by the obese (ob) gene and, since its discovery by Friedman, it has been linked to obesity [2].

Despite the action of leptin in energy homeostasis being known to be mainly a long-term control, by modulating the hypothalamic circuits that regulate feeding and energy expenditure [3], a short-term mediation of the neuroendocrine response to fasting has also been evidenced [4]. Action of leptin at peripheral organs gained strength during the last years [5], including effects at peripheral sensory system [6, 7]. Elevation in plasma leptin concentration has been reported to alter olfactory-mediated responses to food odours, but there is debate on whether it increases [8] or decreases [9–11] responsiveness.

A relation between plasma leptin levels and sweet taste has been proposed by different authors based on studies performed both in humans [12] and in rodents [13]. However, studies are not totally in agreement: whereas some observed decreased responses to the sweet stimulus sucrose [7, 14], others reported an increased responsiveness [15] or no observable effects on sweet taste response [16].

Sweet has been the basic taste mostly linked to leptin effects, with some authors defending that this hormone does not influence the responses for the other basic tastes [17]. However, some researchers reported that concentration of leptin in blood is related to other tastes/oral sensations, apart from sweet: it was observed that regular weight men sensitive for the bitter taste elicited by PTC (phenylthiocarbamide) presented higher plasma leptin levels, comparatively to the nonsensitive pairs [18]; one study reported that, in a low grade inflammation related to obesity, alterations in orosensory perception of oleic acid are related to circulatory levels of leptin [19].

The presence of leptin in saliva is well known. It is present in lower amounts than in blood, but there seems to be a relation between the levels present in both fluids, despite this relation being affected by weight status [20]. The exact function of salivary leptin is not completely understood. At oral cavity level, a physiological role of salivary leptin as a growth factor for keratinocyte proliferation was reported [21]. Recently, Glendinning and colleagues [16] suggested that salivary leptin might act at leptin receptor level present in taste cells, in a way similar to what was reported for the satiation peptide PYY [22]. Moreover, Fábián and colleagues [23], by reviewing the mechanisms of taste perception, hypothesized that leptin, besides having an endocrine action, may act in an exocrine manner, at the level of taste receptor cells. The exact way in which this happens is not known, since there is a lack of knowledge about the position, in basal or apical membrane, of leptin receptors at taste cell level. Nevertheless, if the leptin present in saliva has a direct action in taste perception, it would be expected to have a relationship between the levels at which this hormone is present in saliva and taste response. From our knowledge, such relationship has not been tested so far.

In this pilot study, we aimed at assessing whether there is a link between salivary leptin levels and the perception of two basic tastes (sweet and bitter) and if existing, whether BMI may affect it.

## 2. Material and Methods

### 2.1. Participants

Children from the third year of the 1st cycle of basic education (9-10 years old) from the schools of Evora, Alentejo Region, Portugal, were recruited (*N* = 121; 60 boys and 61 girls). Tests were performed in the morning and children were advised not to eat nor drink anything different from water 1 : 30 hours before testing. Anthropometric parameters (height and weight) were collected at the beginning of the sessions. Height was measured using a stature meter. Weight was measured using an electronic weighing machine to nearest 0.1 kg [24]. Children were classified according to World Health Organization (WHO) that defines overweight (preobesity + obesity) when Body Mass Index (BMI) is equal or superior to +1 standard deviation of reference median, equivalent to 85th percentile and coincident to BMI of 25 kg/m2 of adult age. For analysis, preobese + obese were included in one unique group referred to as “overweight.”

For taste tests and saliva collection, parents or tutors were informed of the procedures and objectives of the study and gave their written informed consent. All procedures were in accordance with the* Declaration of Helsinki* for Medical Research involving Human Subjects and were approved by the institutional ethical committee, from the University of Évora and from Alentejo Regional Health Authority.

### 2.2. Taste Response Tests

For assessment of sweet taste response, sucrose thresholds were assessed and the protocol tested by Knof and colleagues [25] was adopted. Briefly, tests were carried out as a game, in which children were detectives and searched for the solutions that differed in taste from water. Solutions, prepared with distilled water, were presented by ascendant order of concentration (3.0, 4.0, 8.0, 12.0, and 16.0 g/L). Individuals received also a cup of distilled water (the same type of water used to prepare each stimulus solution) and were advised to compare each test solution with this water. The lower stimuli concentration detected as differing from water was considered the threshold. This was only considered when all the other higher concentrations were also detected as differing from water.

Bitter taste perception was assessed by determining caffeine taste thresholds using a procedure similar to the one described for sweet taste. The solutions presented contained the following concentrations of caffeine: 0.15, 0.20, 0.25, 0.3, and 0.4 mg/mL. Sweet and bitter taste tests were performed in the same period, in a randomized order.

Children were classified, according to their caffeine and sucrose detection thresholds in sensitivity groups for bitter and sweet tastes, respectively. If their threshold detection was below the median threshold concentration of the full sample, it was considered as sensitive; otherwise it was low sensitive, similarly to the criterion used by IDEFICS consortium [26].

### 2.3. Saliva Collection and Salivary Leptin Quantification

Before taste tests, unstimulated whole saliva was collected using the drooling technique. Each of the children rinsed their mouth with water and was asked to swallow to remove water and saliva from the mouth. Each of the children received instructions to drain all the saliva produced during 4 minutes to a tube maintained on ice. Saliva obtained was frozen at −20°C. After that, samples were thawed on ice and centrifuged at 13,000*g* for 30 min, at 4°C, to remove mucinous and insoluble material. Supernatant was divided into aliquots and frozen at −20°C until analysis.

For salivary leptin measurement, by enzyme-linked immunosorbent assay (ELISA), samples were thawed at room temperature. A commercial ELISA kit for human leptin (RayBio®, ELH-Leptin-1) was used and the assay was conducted according to the manufacturer's instructions: intra-assay variation was 1.4–8.6%. Each sample from each individual was run in duplicate. Samples were analyzed without dilution.

### 2.4. Statistical Analysis

All data were analyzed using descriptive statistics, and normality and homoscedasticity were evaluated using the Kolmogorov-Smirnoff and Levene tests, respectively.

For classification in groups of sweet taste sensitivity or bitter children taste sensitivity, the median of each of the taste thresholds was calculated. Individuals were considered sensitive if the threshold was lower than the median or low sensitive if it was equal or higher. For comparison among taste sensitivity groups, independent *t*-test was performed. Statistical significance was considered for *P* < 0.05. All statistical analysis procedures were performed using the SPSS 21.0 software package (SPSS Inc., Chicago, USA).

## 3. Results

### 3.1. Salivary Leptin Levels in Relation to Age, Sex, and Body Mass Index (BMI)

No major differences in salivary leptin levels were observed between sexes (10.41 ± 0.76 versus 9.28 ± 0.63 (*P* = 0.255), boys and girls, resp.).

**Figure 1:**
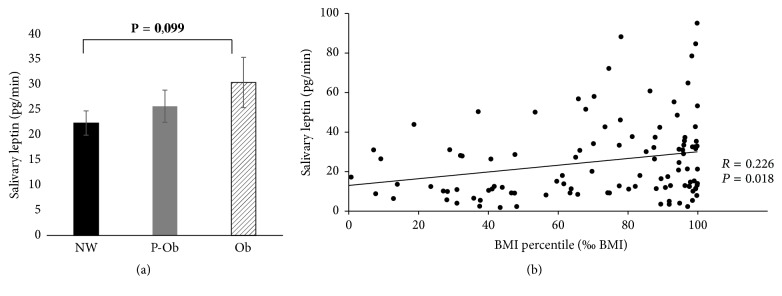
Salivary leptin levels according to ‰ BMI [(a) leptin (pg/min) mean ± standard error for normal weight (NW), preobese (P-Ob), and obese (Ob) children; (b) Spearman correlation between salivary leptin and ‰ BMI].

Concerning BMI percentile (‰ BMI), salivary leptin concentration (pg/mL) did not differ between normal weight, preobese, and obese children. However, taking into consideration the individual salivary flow rate, the amount of leptin secreted per unit of time (pg/min) is positively correlated with ‰ BMI, despite the correlation being weak (*R* = 0.226; *P* = 0.018), with a tendency for higher salivary leptin levels in obese children, comparatively to normal weight ones (Figure 1).

### 3.2. Salivary Leptin Levels in Relation to Taste Perception

Based on the classification criteria reported in the Material and Methods, a total of 50 sweet sensitive and 68 sweet low sensitive children were evaluated. Regarding caffeine detection thresholds, the numbers of children tested were 55 sensitive and 57 low sensitive ones.

**Figure 2:**
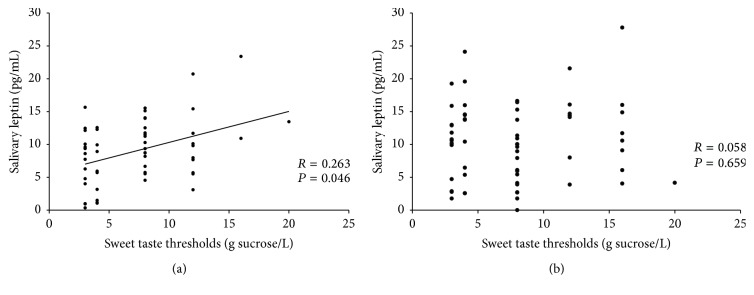
Correlation between sucrose thresholds and saliva leptin levels in girls (a) and boys (b); sweet taste thresholds correspondent to recognition of sucrose concentrations of 3.0, 4.0, 8.0, 12.0, and 16.0 g/L.

Spearman's correlation coefficient revealed a positive correlation between sucrose thresholds and salivary leptin concentration (pg/mL) in girls (*R* = 0.263; *P* = 0.046; *N* = 58), whereas in boys such a correlation was not observed (*R* = 0.058; *P* = 0.659; *P* = 60) (Figure 2).

**Figure 3:**
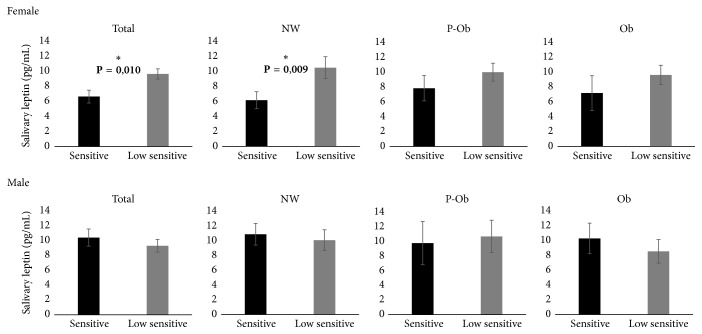
Salivary leptin levels in children with different sweet taste sensitivity [NW: normal weight (*N* = 30 girls and 34 boys); P-Ob: preobese (*N* = 16 girls and 15 boys); Ob: obese (*N* = 12 girls and 13 boys)].

Taking into account the results from Section 3.1, indicating a tendency for an effect of weight status in salivary leptin levels, taste sensitivity groups were compared through ANCOVA, using ‰ BMI as covariate. Differences were observed only in girls, for which higher levels of salivary leptin were observed in the ones low sensitive for the sweet taste of sucrose [6.98 ± 0.90 versus 10.14 ± 0.72 pg/mL (*P* = 0.010), sensitive and low sensitive, resp.]. Boys with different sweet taste sensitivities presented no differences in salivary leptin levels [10.80 ± 1.21 versus 9.65 ± 0.88 pg/mL (*P* = 0.424), sensitive and low sensitive, resp.]. Interestingly, the differences in leptin concentration (pg/mL) between sweet taste sensitivity groups were only statistically significant in normal weight girls [6.45 ± 1.19 versus 11.01 ± 1.53 (*P* = 0.009), sensitive and low sensitive, resp.], being not significant either in preobese [8.14 ± 1.77 versus 10.39 ± 1.25 (*P* = 0.142), sensitive and low sensitive, resp.] or in obese girls [7.46 ± 2.42 versus 9.99 ± 1.35 (*P* = 0.399), sensitive and low sensitive, resp.] (Figure 3).

**Figure 4:**
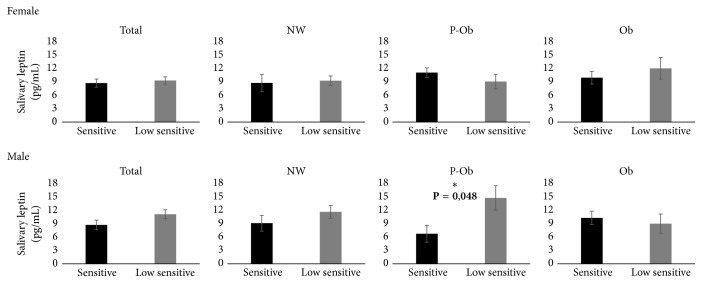
Salivary leptin levels in children with different bitter taste sensitivity (NW – normal weight; P-Ob: preobese; Ob: obese). [NW: normal weight (*N* = 28 girls and 31 boys; P-Ob: preobese *N* = 16 girls and 13 boys); Ob: obese (*N* = 13 girls and 13 boys)].

Salivary leptin levels were also observed to be related to bitter taste sensitivity, but, in opposition to sweet taste, this was observed only in boys. And, similarly to sweet taste, ‰ BMI affects the relationship between salivary leptin concentration and bitter taste sensitivity. A higher concentration of salivary leptin was observed in individuals with lower sensitivity for bitter taste, but only in preobese male. No differences were observed in girls (Figure 4).

## 4. Discussion

The role of leptin in the regulation of food intake at the central nervous system (CNS) level has been extensively studied [27]; however, the activity of this hormone at peripheral level is not completely understood. In 2001, Gröschl and colleagues [20] identified, for the first time, the presence of leptin in human saliva [20]. From then on, different assumptions about the implications of the salivary hormone in food perception have been reported. Although changes in plasmatic leptin levels have been suggested as potentially involved in sweet taste perception [17], to our knowledge, similar studies have not been performed at salivary level. The present study was designed to test the hypothesis that salivary leptin levels are related to taste perception in children. Based on that, salivary leptin levels from children with differences in bitter and sweet tastes sensitivities were compared. Moreover, the potential effect of overweight/obesity in salivary leptin-taste relationship was investigated.

In the present study we did not observe statistically significant differences between sexes. Although leptin in blood has been reported to be higher in females than in males [28], for the levels of this hormone in saliva there are conflicting outcomes: some authors report higher salivary leptin levels in females, comparatively to males, either in adults [29] or in young healthy subjects [30], whereas others, in line with our results, observed no differences [31, 32].

Concerning BMI, plasmatic leptin levels are reported to be correlated with BMI, both in children [33] and in adults [34]. However, for the levels of this hormone in saliva studies are not consensual and whereas some authors report salivary leptin as being correlated with BMI [29], others do not [32]. In the present study, despite no statistically significant differences, a tendency for higher levels of leptin in obese people was observed. This difference exists only if salivary flow rate is taken into consideration, that is, if looking for total amounts of salivary leptin secreted per unit of time. Gröschl and colleagues [20] observed that salivary leptin levels are influenced by saliva stimulation: when saliva flow rate increases due to stimulation leptin concentration decreases but total leptin increases.

An interesting finding of the present work is that salivary leptin relation with taste perception is not the same for different basic tastes, nor sexes. For sweet taste, a relationship between salivary leptin concentration and sensitivity was evident in girls, with the low sensitive presenting significantly higher salivary leptin levels. The influence of leptin at the level of sweet taste perception has been considered in the last years, despite some conflicting results [7, 15]. In a recent study [16] increases in plasma leptin levels did not induce changes in sweet taste responses. Although these authors confirmed the expression of leptin receptors in taste cells, they hypothesized that these receptors in taste cells might be not sufficiently accessible to circulating leptin and that the leptin effect in sweet taste might be through a paracrine route, possibly involving leptin present in saliva. Although, from our knowledge, there are no reports about the exact location, in basal or apical membrane, of leptin receptor at taste cell membrane level, the hypothesis of its presence at apical level, with the possibility of access by leptin in saliva, can be considered, similarly to what has been observed for other receptors for gastrointestinal hormones involved in satiety control, such as peptide YY (PYY) [22]. In this last case, PYY present in saliva appears to modulate taste perception through direct interaction with its receptors in taste cells. Moreover, the existence of a pronounced permeability barrier in taste buds limits the access of peptides and hormones to taste buds through circulation [35], which makes it more probable that the action of hormones in taste can be, at least partially, through saliva. Further studies need to be performed to elucidate leptin receptor location and direct action of salivary leptin.

The reason why a relationship between salivary leptin levels and sweet taste sensitivity exists only in girls needs to be further explored. Probably, an effect of sexual hormones may be an explanation. Although the children participating in this study were 9-10-year-old, there are evidences that girls have higher levels of estrogen than boys, even before pubertal signals have appeared [36, 37], and this cannot be excluded in our case. The theory of a modulation of the influence of salivary leptin in sweet taste perception by sexual hormones can be reinforced based on the evidences emerging from literature: (1) an action of estrogen at sweet taste level has already been reported [38]; (2) some different studies mention that estrogen induce increases in leptin sensitivity at receptor level, namely, at hypothalamus, where leptin and estrogen cooperate in the regulation of energy homeostasis [39, 40] or in peripheral tissues, where an interaction between leptin and estrogen has also been reported [41]; (3) a recent study suggested that leptin effect in sweet taste preference is affected by sexual hormones, as observed by different relationship between circulating leptin levels and sweet food preference in the different phases of menstrual cycle [42].

Another interesting question raised by our results is “why, in girls, salivary leptin levels relate with sweet taste sensitivity only in normal weight individuals?” In fact, the statistically significant differences between sweet sensitive and low sensitive girls were not obtained for preobese or obese girls. One possible explanation that should be further explored in subsequent studies is the existence of some leptin resistance in preobese and obese individuals. Leptin receptor signaling cascade appears to be compromised with obesity, resulting in the lack of action of the high levels of this hormone in obese people [43]. This is relatively well known at hypothalamus levels but less well studied at systemic level. If salivary leptin acts at taste receptor cell level, inhibiting sweet taste detection, leptin resistance at this level, in preobese and obese children, would result in a lack of effect of salivary leptin in sweet taste perception, in accordance with our results.

Concerning bitter taste, although there were no differences between girls with different sensitivities, in the mean salivary leptin concentrations, in boys these were higher in children low sensitive, although only in the ones preobese. Until now, the relationship between leptin levels and bitter taste presents no consensus. Some authors observing an effect of blood leptin in sweet taste sensitivity failed to observe similar effect at bitter taste level [17]. However, a study reported that adult males who perceived PROP bitter taste with higher intensity presented higher circulating levels of this hormone [18], supporting a possible involvement of leptin also in the perception of this basic taste. In the present study, significantly higher salivary leptin levels in normal weight boys that were sensitive for bitterness were not observed. Curiously, in preobese boys, the opposite was observed, with low sensitive individuals presenting higher levels of leptin in saliva. The different results for normal weight and overweight individuals can be hypothesized to be done to some leptin resistance, similarly to what has been referred above. However, the meaning of this difference needs to be further deeply investigated.

A possible effect of leptin in both sweet and bitter tastes can be understandable by the relevance that these tastes have as drivers for ingestion: sweet taste motivates ingestion and bitter taste acts as deterrent of intake [44]. Since leptin is a hormone involved in satiety [45], it could be expected that higher salivary leptin concentration induced decreases in the perception level of pleasant signals and increases in sensitivity for unpleasant sensations. This was observed for sweet taste perception, but not for bitterness.

## 5. Conclusions

Our findings indicate a relationship between salivary leptin levels and taste perception. To our knowledge, this is the first time that the levels of leptin in saliva are related to taste perception. Until now, the studies aimed at investigating the effects of this hormone at taste receptor level were in vitro or, in the case of the ones in vivo, have been focused only in the levels of leptin in blood. However, recent findings point to a possible action of salivary leptin at taste level: on one hand, a relationship between blood leptin levels and sweet taste sensitivity is not always observed; on the other hand, the presence of a pronounced permeability barrier in taste buds limits the access of hormones from blood through a basolateral route.

Our novel findings extend the discussion on how salivary hormones may affect taste perception and signals to the need of studies about how salivary hormones may act to modulate taste responses.
